# 4-Benz­yloxy-3-methoxy­benzonitrile

**DOI:** 10.1107/S1600536809005613

**Published:** 2009-02-21

**Authors:** Muhammad Hanif, Muhammad Rafiq, Muhammad Saleem, Ghulam Qadeer, Wai-Yeung Wong

**Affiliations:** a Department of Chemistry, Quaid-i-Azam Univeristy, Islamabad 45320, Pakistan; b Department of Chemistry, University of Sargodah, Sargodah, Pakistan; c Department of Chemistry, Hong Kong Baptist University, Waterloo Road, Kowloon Tong, Hong Kong, People’s Republic of China

## Abstract

In the mol­ecule of the title compound, C_15_H_13_NO_2_, the aromatic rings are oriented at a dihedral angle of 81.65 (3)°. In the crystal structure, weak inter­molecular C—H⋯N hydrogen bonds link the mol­ecules into chains along the *b* axis.

## Related literature

For the potential application of highly conjugated mol­ecules in nanoelectronics, see: Tour (2003 ▶) and in optoelectronics, see: Lind *et al.* (2004 ▶); Ornelas *et al.* (2005 ▶, 2008 ▶). Terminal cyano groups provide the ability to coordinate to transition metal centres such as RuCp, see: Garcia *et al.* (2001 ▶); Ornelas *et al.* (2005 ▶). For bond-length data, see: Allen *et al.* (1987 ▶).
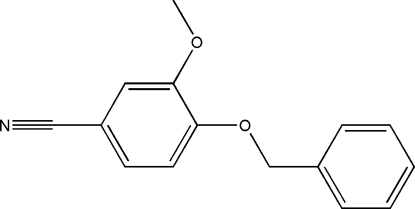


## Experimental

### 

#### Crystal data

C_15_H_13_NO_2_
*M* *_r_* = 239.26
Monoclinic, 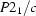

*a* = 14.9434 (12) Å
*b* = 9.5469 (8) Å
*c* = 8.8522 (7) Å
β = 102.663 (2)°
*V* = 1232.16 (17) Å^3^
*Z* = 4
Mo *K*α radiation
μ = 0.09 mm^−1^
*T* = 173 K
0.32 × 0.25 × 0.23 mm

#### Data collection

Bruker SMART CCD area-detector diffractometer
Absorption correction: multi-scan (*SADABS*; Bruker, 2001 ▶) *T*_min_ = 0.864, *T*_max_ = 0.980
7286 measured reflections
2983 independent reflections
2499 reflections with *I* > 2σ(*I*)
*R*_int_ = 0.018

#### Refinement

*R*[*F* ^2^ > 2σ(*F* ^2^)] = 0.038
*wR*(*F* ^2^) = 0.114
*S* = 1.02
2983 reflections
163 parameters
H-atom parameters constrained
Δρ_max_ = 0.23 e Å^−3^
Δρ_min_ = −0.17 e Å^−3^

### 

Data collection: *SMART* (Bruker, 2001 ▶); cell refinement: *SAINT* (Bruker, 2002 ▶); data reduction: *SAINT*; program(s) used to solve structure: *SHELXS97* (Sheldrick, 2009 ▶); program(s) used to refine structure: *SHELXL97* (Sheldrick, 2008 ▶); molecular graphics: *ORTEP-3 for Windows* (Farrugia, 1997 ▶); software used to prepare material for publication: *SHELXTL* (Sheldrick, 2009 ▶).

## Supplementary Material

Crystal structure: contains datablocks I, global. DOI: 10.1107/S1600536809005613/hk2626sup1.cif

Structure factors: contains datablocks I. DOI: 10.1107/S1600536809005613/hk2626Isup2.hkl

Additional supplementary materials:  crystallographic information; 3D view; checkCIF report

## Figures and Tables

**Table 1:** Hydrogen-bond geometry (Å, °)

*D*—H⋯*A*	*D*—H	H⋯*A*	*D*⋯*A*	*D*—H⋯*A*
C14—H14*B*⋯N1^i^	0.98	2.58	3.5170 (17)	160

Symmetry code: (i) 
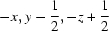
.

**Fig. 1.**
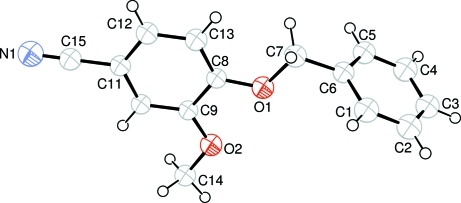
The molecular structure of the title molecule, with the atom-numbering scheme.

**Fig. 2.**
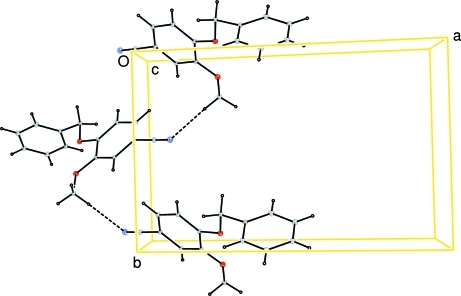
A partial packing diagram of the title compound. Hydrogen bonds are shown as dashed lines.

**Fig. 3.**
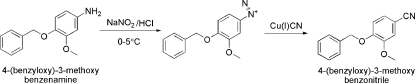
The formation of the title compound.

## supplementary crystallographic information

### Comment

Schiff base compounds have attracted great attention for many years. They play an important role in the development of coordination chemistry related to catalysis and enzymatic reactions, magnetism, photochromism and thermochromism. We report herein the crystal structure of the title compound.

In the title compound (Fig. 1), the bond lengths (Allen *et al.*, 1987) and angles are within normal ranges. Rings A (C1-C6) and B (C8-C13) are, of course, planar, and they are oriented at a dihedral angle of 81.65 (3)°.

In the crystal structure, weak intermolecular C-H···N hydrogen bonds (Table 1) link the molecules into chains along the b axis (Fig. 2), in which they may be effective in the stabilization of the structure.

The preparation of highly conjugated molecules has been of great interest for their potential applications in fields such as nanoelectronics (Tour, 2003) or optoelectronics (Ornelas *et al.*,  2005, 2008; Lind *et al.*,  2004). Terminal cyano groups provide the ability to coordinate to transition metal centres such as RuCp (Cp = cyclopentadienyl); (Garcia *et al.*,  2001; Ornelas *et al.*,  2005) which should result in an increase of the physical properties such as the first molecular hyperpolarizability β, which is reported to rise with the coordination to cyclopentadienylruthenium type centres (Ornelas *et al.*,  2005, 2008). As such the preparation of the π-conjugated title compound was intended for the preparation of dinuclear ruthenium complexes for nanoelectronic application.

### Experimental

For the preparation of the title compound, 4-(benzyloxy)-3-methoxy benzenamine (2.29 g, 10 mmol) was treated with sodium nitrite (0.7 g, 10 mmol) in the presence of concentrated hydrochloric acid (10 ml) at 273-278 K. Aqueous cupreous cyanate solution (48%, 1.05 g, 10 mmol) was added into the resulting diazonnium salt (1.95 g, 8 mmol). The obtained title compound was separated and recrystallized in ethanol/THF mixture (yield; 65%, m.p. 411-412 K).

### Refinement

H atoms were positioned geometrically, with C-H = 0.95, 0.99 and 0.98 Å for aromatic, methylene and methyl H, respectively, and constrained to ride on their parent atoms, with U_iso_(H) = xU_eq_(C), where x = 1.5 for methyl H and x = 1.2 for all other H atoms.

### Crystal data

**Table:**

C_15_H_13_NO_2_	*F*(000) = 504
*M**_r_* = 239.26	*D*_x_ = 1.290 Mg m^−^^3^
Monoclinic, *P*2_1_/*c*	Mo *K*α radiation, λ = 0.71073 Å
Hall symbol: -P 2ybc	Cell parameters from 7856 reflections
*a* = 14.9434 (12) Å	θ = 2.6–28.3°
*b* = 9.5469 (8) Å	µ = 0.09 mm^−^^1^
*c* = 8.8522 (7) Å	*T* = 173 K
β = 102.663 (2)°	Block, colorless
*V* = 1232.16 (17) Å^3^	0.32 × 0.25 × 0.23 mm
*Z* = 4	

### Data collection

**Table:**

Bruker SMART CCD area-detector diffractometer	2983 independent reflections
Radiation source: fine-focus sealed tube	2499 reflections with *I* > 2σ(*I*)
graphite	*R*_int_ = 0.018
ω and φ scans	θ_max_ = 28.3°, θ_min_ = 2.6°
Absorption correction: multi-scan (*SADABS*; Bruker, 2001)	*h* = −17→19
*T*_min_ = 0.864, *T*_max_ = 0.980	*k* = −11→12
7286 measured reflections	*l* = −11→11

### Refinement

**Table:**

Refinement on *F*^2^	Primary atom site location: structure-invariant direct methods
Least-squares matrix: full	Secondary atom site location: difference Fourier map
*R*[*F*^2^ > 2σ(*F*^2^)] = 0.038	Hydrogen site location: inferred from neighbouring sites
*wR*(*F*^2^) = 0.114	H-atom parameters constrained
*S* = 1.02	*w* = 1/[σ^2^(*F*_o_^2^) + (0.0606*P*)^2^ + 0.2089*P*] where *P* = (*F*_o_^2^ + 2*F*_c_^2^)/3
2983 reflections	(Δ/σ)_max_ < 0.001
163 parameters	Δρ_max_ = 0.23 e Å^−^^3^
0 restraints	Δρ_min_ = −0.17 e Å^−^^3^

### Special details

**Table:**

Geometry. All e.s.d.'s (except the e.s.d. in the dihedral angle between two l.s. planes) are estimated using the full covariance matrix. The cell e.s.d.'s are taken into account individually in the estimation of e.s.d.'s in distances, angles and torsion angles; correlations between e.s.d.'s in cell parameters are only used when they are defined by crystal symmetry. An approximate (isotropic) treatment of cell e.s.d.'s is used for estimating e.s.d.'s involving l.s. planes.
Refinement. Refinement of *F*^2^ against ALL reflections. The weighted *R*-factor *wR* and goodness of fit *S* are based on *F*^2^, conventional *R*-factors *R* are based on *F*, with *F* set to zero for negative *F*^2^. The threshold expression of *F*^2^ > σ(*F*^2^) is used only for calculating *R*-factors(gt) *etc*. and is not relevant to the choice of reflections for refinement. *R*-factors based on *F*^2^ are statistically about twice as large as those based on *F*, and *R*- factors based on ALL data will be even larger.

### Fractional atomic coordinates and isotropic or equivalent isotropic displacement parameters (Å^2^)

**Table:**

	*x*	*y*	*z*	*U*_iso_*/*U*_eq_	
O1	0.26381 (5)	0.56473 (9)	0.00751 (9)	0.0409 (2)	
O2	0.26362 (5)	0.38653 (9)	0.22449 (9)	0.0423 (2)	
N1	−0.09205 (7)	0.55876 (14)	0.35630 (13)	0.0545 (3)	
C1	0.44027 (9)	0.66914 (15)	−0.09352 (15)	0.0492 (3)	
H1A	0.4473	0.7296	−0.0065	0.059*	
C2	0.51719 (9)	0.62309 (16)	−0.14277 (16)	0.0520 (3)	
H2A	0.5765	0.6523	−0.0893	0.062*	
C3	0.50825 (9)	0.53599 (14)	−0.26781 (15)	0.0479 (3)	
H3A	0.5612	0.5033	−0.3000	0.058*	
C4	0.42195 (9)	0.49571 (15)	−0.34716 (16)	0.0510 (3)	
H4A	0.4155	0.4364	−0.4351	0.061*	
C5	0.34464 (8)	0.54158 (13)	−0.29886 (14)	0.0440 (3)	
H5A	0.2855	0.5137	−0.3543	0.053*	
C6	0.35295 (8)	0.62749 (12)	−0.17068 (13)	0.0375 (2)	
C7	0.26977 (8)	0.66610 (13)	−0.11153 (14)	0.0425 (3)	
H7A	0.2760	0.7620	−0.0680	0.051*	
H7B	0.2141	0.6623	−0.1961	0.051*	
C8	0.18984 (7)	0.57131 (11)	0.07225 (12)	0.0339 (2)	
C9	0.18915 (7)	0.47256 (11)	0.19135 (12)	0.0328 (2)	
C10	0.11690 (7)	0.46987 (12)	0.26518 (12)	0.0343 (2)	
H10A	0.1166	0.4048	0.3462	0.041*	
C11	0.04377 (7)	0.56410 (12)	0.21963 (12)	0.0358 (2)	
C12	0.04428 (8)	0.66074 (13)	0.10355 (13)	0.0403 (3)	
H12A	−0.0055	0.7241	0.0736	0.048*	
C13	0.11769 (8)	0.66500 (13)	0.03071 (13)	0.0399 (3)	
H13A	0.1185	0.7324	−0.0479	0.048*	
C14	0.26552 (8)	0.28301 (13)	0.34102 (13)	0.0411 (3)	
H14A	0.3220	0.2280	0.3534	0.062*	
H14B	0.2124	0.2210	0.3105	0.062*	
H14C	0.2635	0.3288	0.4393	0.062*	
C15	−0.03198 (7)	0.55965 (13)	0.29606 (13)	0.0411 (3)	

### Atomic displacement parameters (Å^2^)

**Table:**

	*U*^11^	*U*^22^	*U*^33^	*U*^12^	*U*^13^	*U*^23^
O1	0.0369 (4)	0.0459 (5)	0.0439 (4)	0.0084 (3)	0.0176 (3)	0.0109 (3)
O2	0.0311 (4)	0.0469 (5)	0.0509 (5)	0.0104 (3)	0.0132 (3)	0.0138 (4)
N1	0.0373 (5)	0.0777 (8)	0.0499 (6)	0.0106 (5)	0.0128 (4)	0.0015 (5)
C1	0.0493 (7)	0.0525 (7)	0.0454 (6)	−0.0055 (5)	0.0094 (5)	−0.0083 (5)
C2	0.0378 (6)	0.0625 (8)	0.0544 (7)	−0.0090 (6)	0.0071 (5)	−0.0007 (6)
C3	0.0408 (6)	0.0529 (7)	0.0547 (7)	−0.0047 (5)	0.0204 (5)	0.0033 (6)
C4	0.0481 (7)	0.0573 (8)	0.0528 (7)	−0.0108 (6)	0.0220 (6)	−0.0127 (6)
C5	0.0383 (6)	0.0496 (7)	0.0463 (6)	−0.0109 (5)	0.0142 (5)	−0.0036 (5)
C6	0.0408 (6)	0.0350 (5)	0.0392 (5)	−0.0020 (4)	0.0139 (4)	0.0061 (4)
C7	0.0475 (6)	0.0398 (6)	0.0435 (6)	0.0055 (5)	0.0171 (5)	0.0084 (5)
C8	0.0317 (5)	0.0370 (5)	0.0337 (5)	0.0025 (4)	0.0085 (4)	−0.0009 (4)
C9	0.0263 (5)	0.0351 (5)	0.0362 (5)	0.0026 (4)	0.0054 (4)	−0.0002 (4)
C10	0.0295 (5)	0.0386 (5)	0.0348 (5)	0.0000 (4)	0.0068 (4)	−0.0003 (4)
C11	0.0293 (5)	0.0427 (6)	0.0354 (5)	0.0022 (4)	0.0072 (4)	−0.0078 (4)
C12	0.0367 (5)	0.0433 (6)	0.0401 (5)	0.0119 (4)	0.0069 (4)	−0.0025 (5)
C13	0.0421 (6)	0.0411 (6)	0.0373 (5)	0.0090 (5)	0.0106 (4)	0.0032 (4)
C14	0.0366 (5)	0.0411 (6)	0.0431 (6)	0.0037 (4)	0.0034 (4)	0.0065 (5)
C15	0.0320 (5)	0.0523 (7)	0.0385 (6)	0.0063 (5)	0.0067 (4)	−0.0043 (5)

### Geometric parameters (Å, °)

**Table:**

C1—C2	1.3872 (18)	C8—C13	1.3870 (15)
C1—C6	1.3916 (17)	C8—C9	1.4161 (15)
C1—H1A	0.9500	C9—O2	1.3624 (12)
C2—C3	1.3673 (19)	C9—C10	1.3794 (14)
C2—H2A	0.9500	C10—C11	1.4046 (14)
C3—C4	1.3810 (18)	C10—H10A	0.9500
C3—H3A	0.9500	C11—C12	1.3822 (16)
C4—C5	1.3878 (17)	C11—C15	1.4411 (14)
C4—H4A	0.9500	C12—C13	1.3897 (15)
C5—C6	1.3834 (16)	C12—H12A	0.9500
C5—H5A	0.9500	C13—H13A	0.9500
C6—C7	1.4967 (15)	C14—O2	1.4244 (13)
C7—O1	1.4478 (13)	C14—H14A	0.9800
C7—H7A	0.9900	C14—H14B	0.9800
C7—H7B	0.9900	C14—H14C	0.9800
C8—O1	1.3535 (12)	C15—N1	1.1402 (15)
			
C8—O1—C7	117.61 (8)	O1—C8—C13	125.25 (10)
C9—O2—C14	117.38 (8)	O1—C8—C9	115.10 (9)
C2—C1—C6	120.51 (12)	C13—C8—C9	119.65 (9)
C2—C1—H1A	119.7	O2—C9—C10	125.03 (9)
C6—C1—H1A	119.7	O2—C9—C8	115.01 (9)
C3—C2—C1	120.43 (12)	C10—C9—C8	119.96 (9)
C3—C2—H2A	119.8	C9—C10—C11	119.51 (10)
C1—C2—H2A	119.8	C9—C10—H10A	120.2
C2—C3—C4	119.72 (12)	C11—C10—H10A	120.2
C2—C3—H3A	120.1	C12—C11—C10	120.68 (10)
C4—C3—H3A	120.1	C12—C11—C15	120.10 (10)
C3—C4—C5	120.20 (12)	C10—C11—C15	119.22 (10)
C3—C4—H4A	119.9	C11—C12—C13	119.87 (10)
C5—C4—H4A	119.9	C11—C12—H12A	120.1
C6—C5—C4	120.58 (11)	C13—C12—H12A	120.1
C6—C5—H5A	119.7	C8—C13—C12	120.33 (10)
C4—C5—H5A	119.7	C8—C13—H13A	119.8
C5—C6—C1	118.55 (11)	C12—C13—H13A	119.8
C5—C6—C7	120.06 (10)	O2—C14—H14A	109.5
C1—C6—C7	121.28 (11)	O2—C14—H14B	109.5
O1—C7—C6	106.16 (9)	H14A—C14—H14B	109.5
O1—C7—H7A	110.5	O2—C14—H14C	109.5
C6—C7—H7A	110.5	H14A—C14—H14C	109.5
O1—C7—H7B	110.5	H14B—C14—H14C	109.5
C6—C7—H7B	110.5	N1—C15—C11	178.73 (13)
H7A—C7—H7B	108.7		

### Hydrogen-bond geometry (Å, °)

**Table:**

*D*—H···*A*	*D*—H	H···*A*	*D*···*A*	*D*—H···*A*
C14—H14B···N1^i^	0.98	2.58	3.5170 (17)	160

Symmetry codes: (i) −*x*, *y*−1/2, −*z*+1/2.
